# Supplementary figures and images for: GranatumX: A Community-engaging, Modularized, and Flexible Webtool for Single-cell Data Analysis
Source: Genomics Proteomics Bioinformatics. 2021 Dec 30;19(3):452–60. doi: 10.1016/j.gpb.2021.07.005 (PMC8864242; doi:10.1016/j.gpb.2021.07.005)

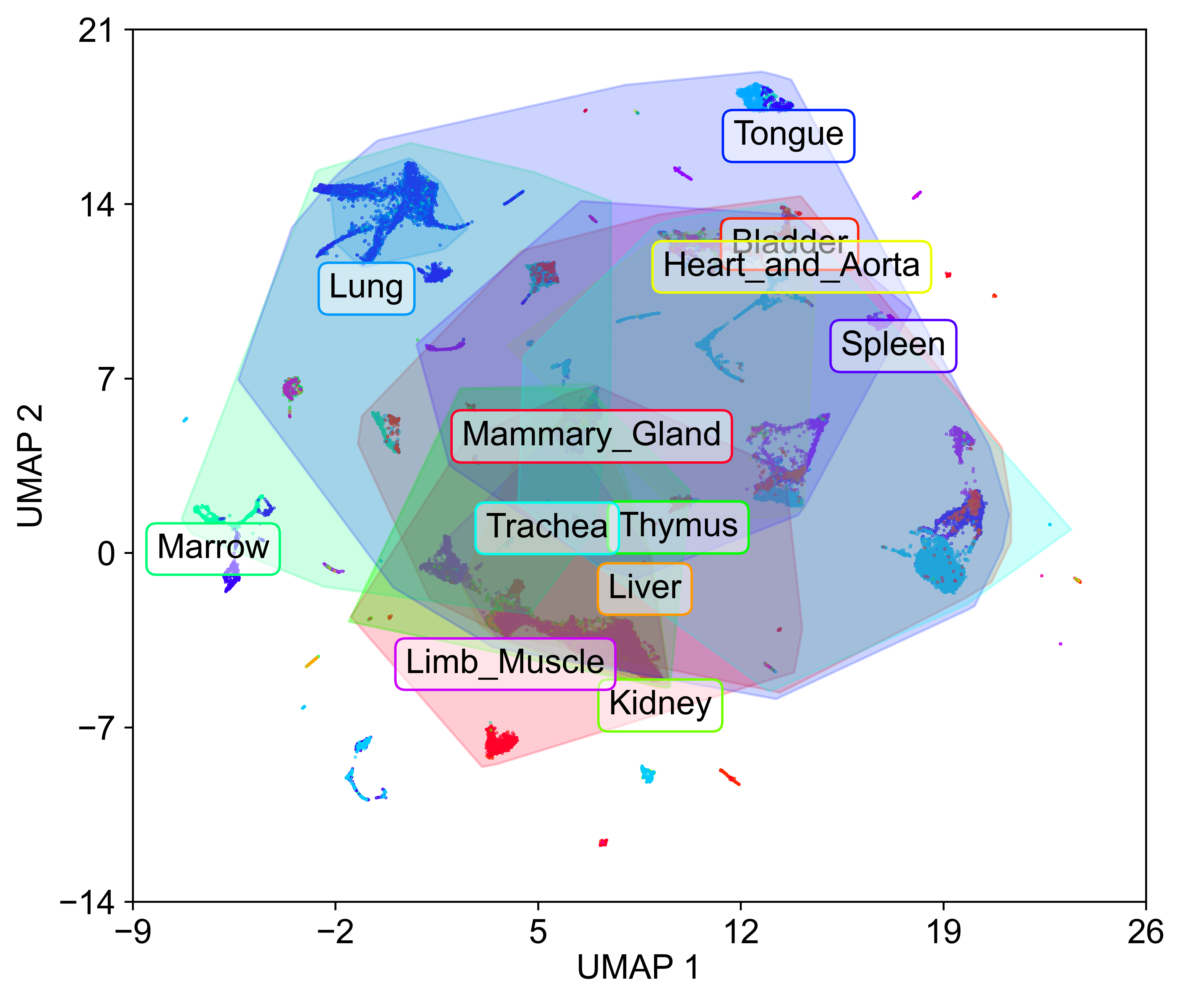

Supplement: Supplementary Figure S1 — UMAP plot with annotated tissue types in the Tabula Muris Consortium data [file mmc1.zip › Figure S1-Au122221.png]
