# Supplementary material for: GranatumX: A Community-engaging, Modularized, and Flexible Webtool for Single-cell Data Analysis
Source: Genomics Proteomics Bioinformatics. 2021 Dec 30;19(3):452–60. doi: 10.1016/j.gpb.2021.07.005 (PMC8864242; doi:10.1016/j.gpb.2021.07.005)
Supplement: Supplementary File S1 — The analysis report using a dataset with metastatic Merkel cell carcinoma from the 10x Genomics platform [file mmc2.pdf]

Supplementary File 1

Analysis Report

This report is generated by GranatumX

This is the pipeline to replicate Supplementary file 1 with GSE117988 data

Upload Files 1.0.0

Assay to upload: **GSE117988_raw.expMatrix_Tumor.csv.zip (12.91 MB)**
File format: **"zip"**
Convert gene IDs: **false**
Species: **"human"**
Convert gene IDs into (HGNC symbol is recommended): **"symbol"**
Add extra info (from BioMart) into gene metadata: **true**
Enter your email address to get notified of any errors encountered in the pipeline: **false**

The assay has **21861** genes (with inferred ID type: Symbol) and **7431** samples.

The first few rows and columns:

```
0.0, 0.0, 0.0, 0.0, 0.0, 0.0, 0.0, 0.0, 0.0, 0.0
1.0, 0.0, 0.0, 0.0, 0.0, 0.0, 0.0, 0.0, 0.0, 0.0
0.0, 0.0, 0.0, 0.0, 0.0, 0.0, 0.0, 0.0, 0.0, 0.0
0.0, 0.0, 0.0, 0.0, 0.0, 0.0, 0.0, 0.0, 0.0, 0.0
0.0, 0.0, 0.0, 0.0, 0.0, 0.0, 0.0, 0.0, 0.0, 0.0
0.0, 0.0, 0.0, 0.0, 0.0, 0.0, 0.0, 0.0, 0.0, 0.0
0.0, 0.0, 1.0, 0.0, 1.0, 0.0, 0.0, 0.0, 0.0, 0.0
0.0, 0.0, 0.0, 0.0, 0.0, 0.0, 0.0, 1.0, 0.0, 0.0
0.0, 0.0, 0.0, 0.0, 0.0, 0.0, 0.0, 0.0, 0.0, 0.0
0.0, 0.0, 0.0, 0.0, 0.0, 0.0, 0.0, 0.0, 0.0, 0.0
```

- Finished upload step in 239.38 seconds*

DeepImpute 2.0.0

Random seed: **12345**
Use automatic gene imputation limit: **true**
Gene rank limit: **2000**
Cell subset: **1**
Assay: **[A]GSE117988_raw.expMatrix_Tumor.csv.zip** (from step 1: Upload Files 1.0.0)

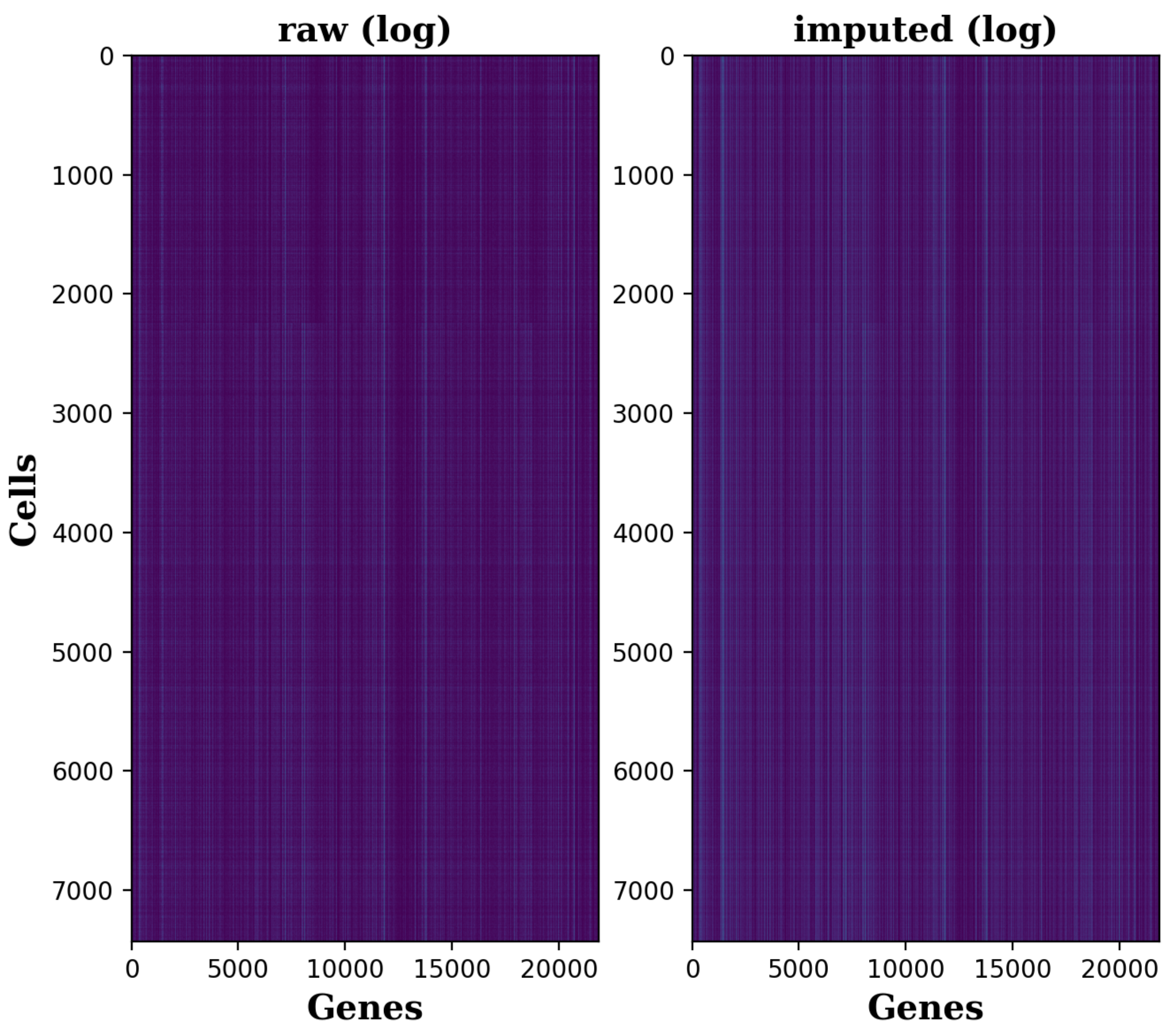

Heatmaps

- Data frame number of rows: **7431**
- Data frame number of columns: **21861**
- Number of imputed genes: **1536**

- Percentage of dropout entries *before* imputation: **92.70%**
- Percentage of dropout entries *after* imputation: **89.11%**
- Accuracy (correlation) on masked data: **0.92**

Cell Normalization 1.0.0

Log transform in the boxplots: **false**
Normalization method: **"quantile"**
Number of cells to plot in the bar-plot: **40**
Assay: **Imputed assay** (from step 2: DeepImpute 2.0.0)

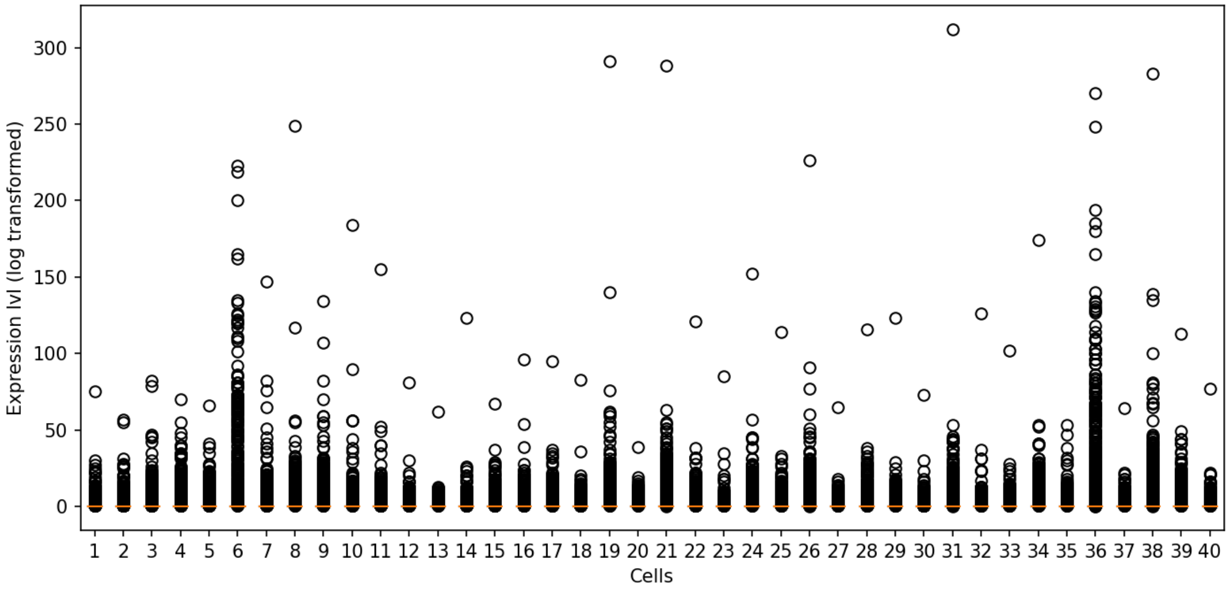

Before normalization: Each bar in the box plot represents one cell.

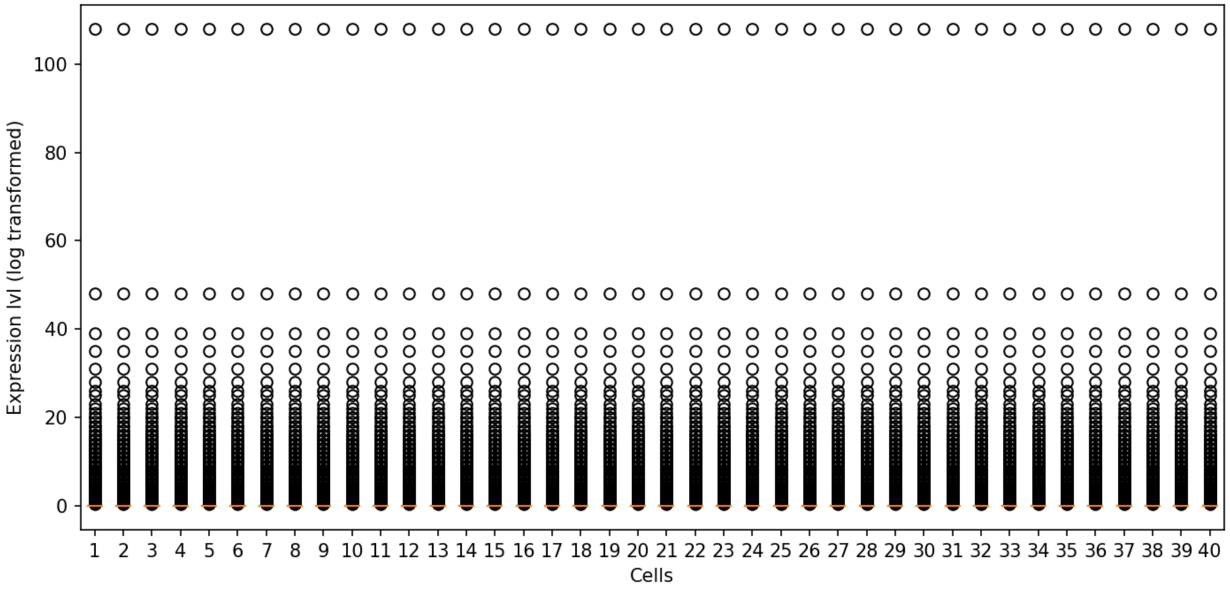

After normalization: Each bar in the box plot represents one cell.

Scanpy Gene Filtering 1.0.0

The gene has to be expressed in at least ___ cells: **3**
The average expression level of the gene has to be greater than: **1**
The average expression level of the gene has to be less than: **999999**
The dispersion of the gene has to be greater than: **0.5**
The dispersion of the gene has to be less than: **999999**
Assay: **Normalized assay** (from step 3: Cell Normalization 1.0.0)

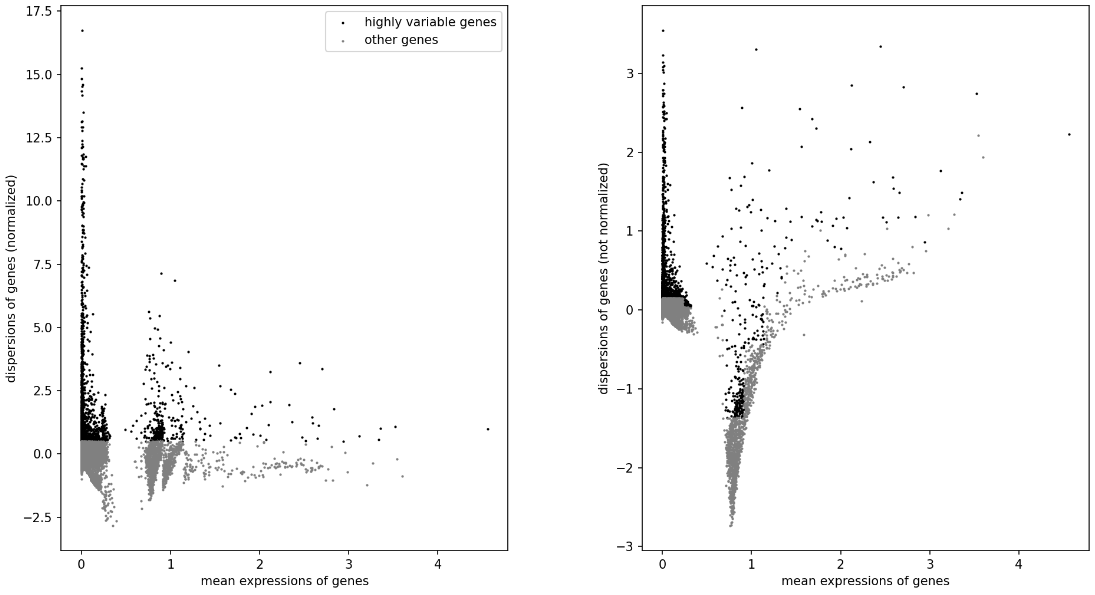

Each dot represent a gene. The gray dots are the removed genes. The x-axis is log-transformed.

Number of genes before filtering: **21861**

Number of genes after filtering: **1594**

Log transformation 1.0.0

The base used for the log function: **2**
The pseudo counts added before log transformation (to avoid getting log(0)): **1**
Assay including matrix and geneIds: **Filtered Assay** (from step 4: Scanpy Gene Filtering 1.0.0)

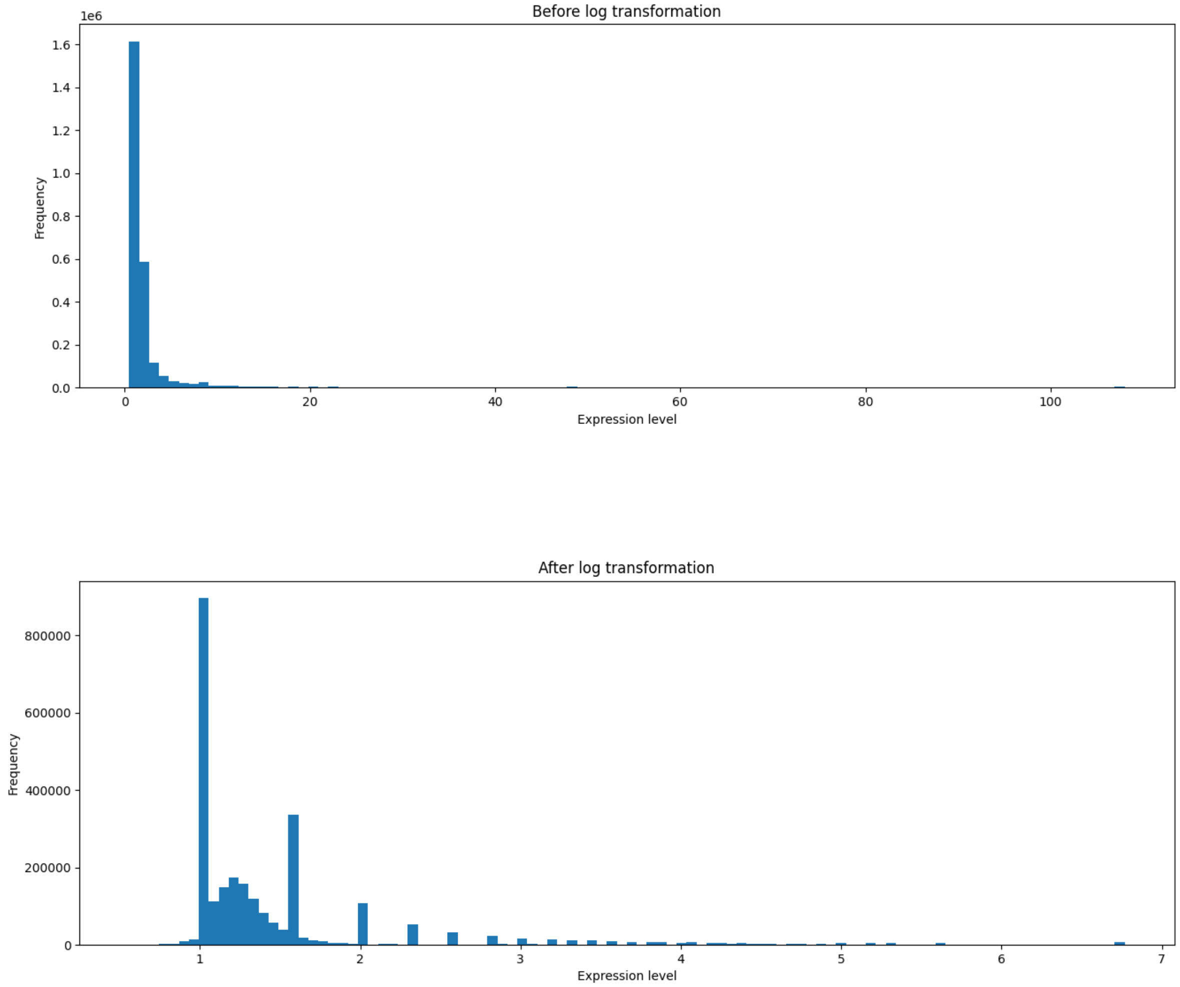

The distribution of expression level before and after log transformation. Only the values greater than the 5 percentile (usually zero in single-cell data) and lower than 95 percentile are considered.

Principal Component Analysis 1.0.0

Number of top components to calculate: **2**

Assay: **Log transformed assay** (from step 5: Log transformation 1.0.0)

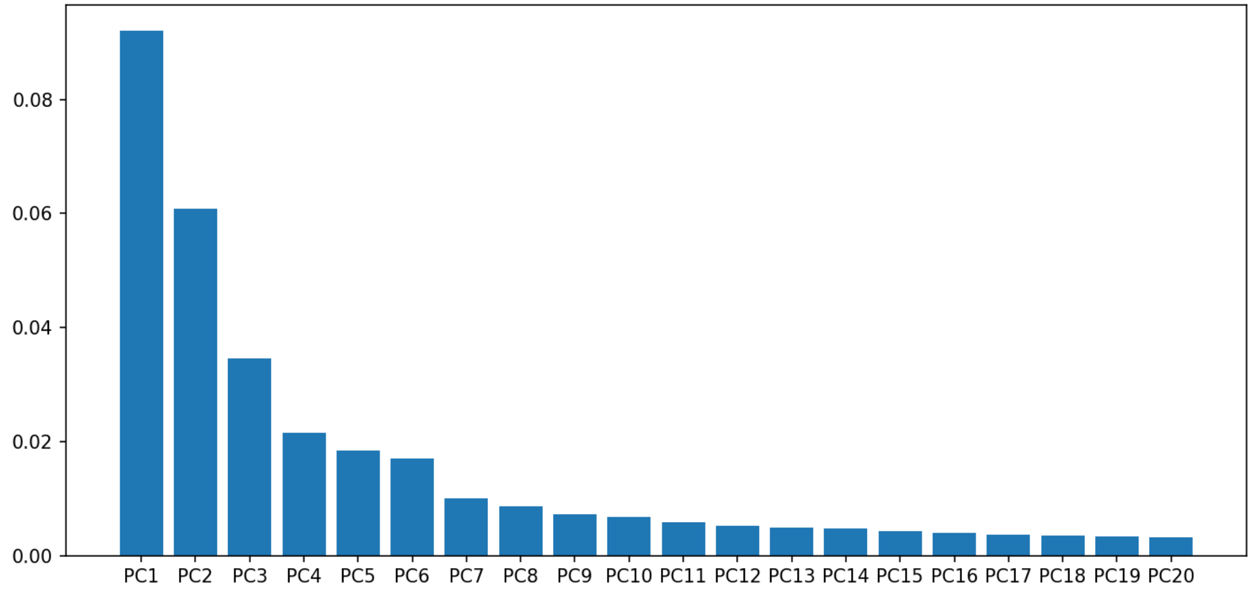

Explained variance (ratio) by each Principal Component (PC)

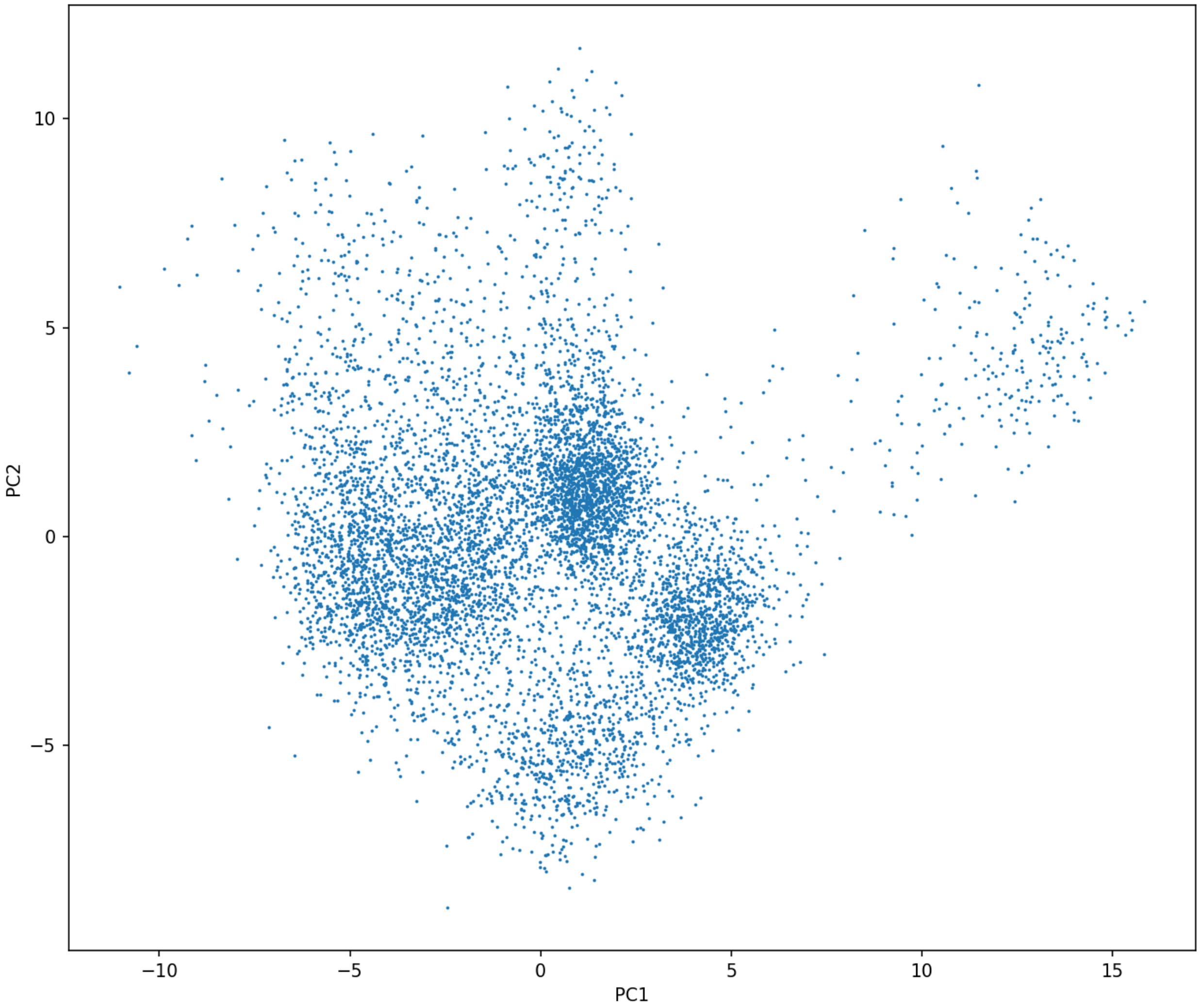

PC1 vs. PC2

t-Distributed Stochastic Neighbor Embedding 1.0.0

Random seed: **56143**

Assay: **Log transformed assay** (from step 5: Log transformation 1.0.0)

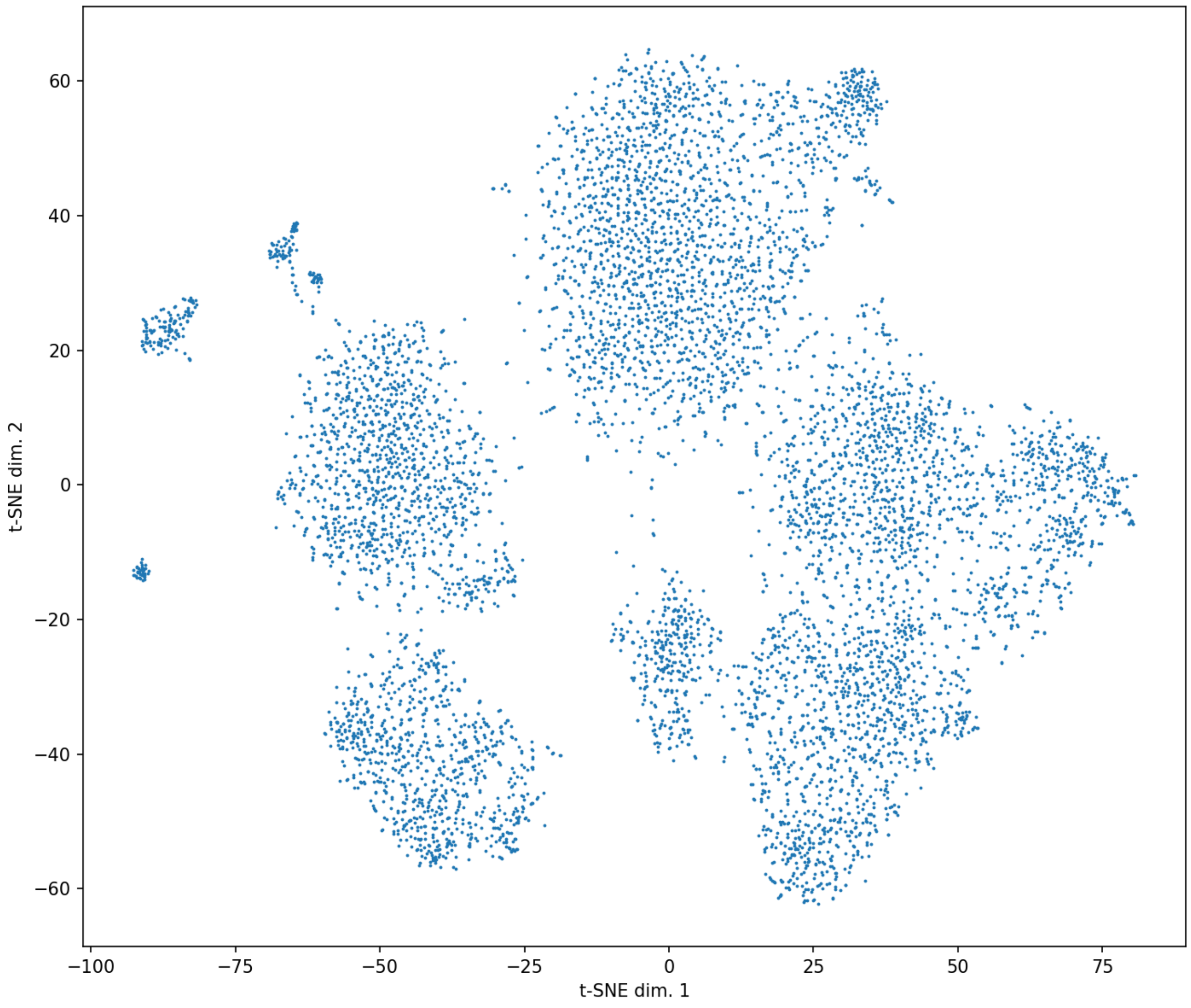

t-SNE plot: each dot represents a cell

UMAP 1.0.0

Number of neighbors (n_neighbors, an integer): **15**
Minimum distance (min_dist, a real number ranges from 0 to 1): **0.1**
Metric (metric): **"euclidean"**
Assay: **Log transformed assay** (from step 5: Log transformation 1.0.0)

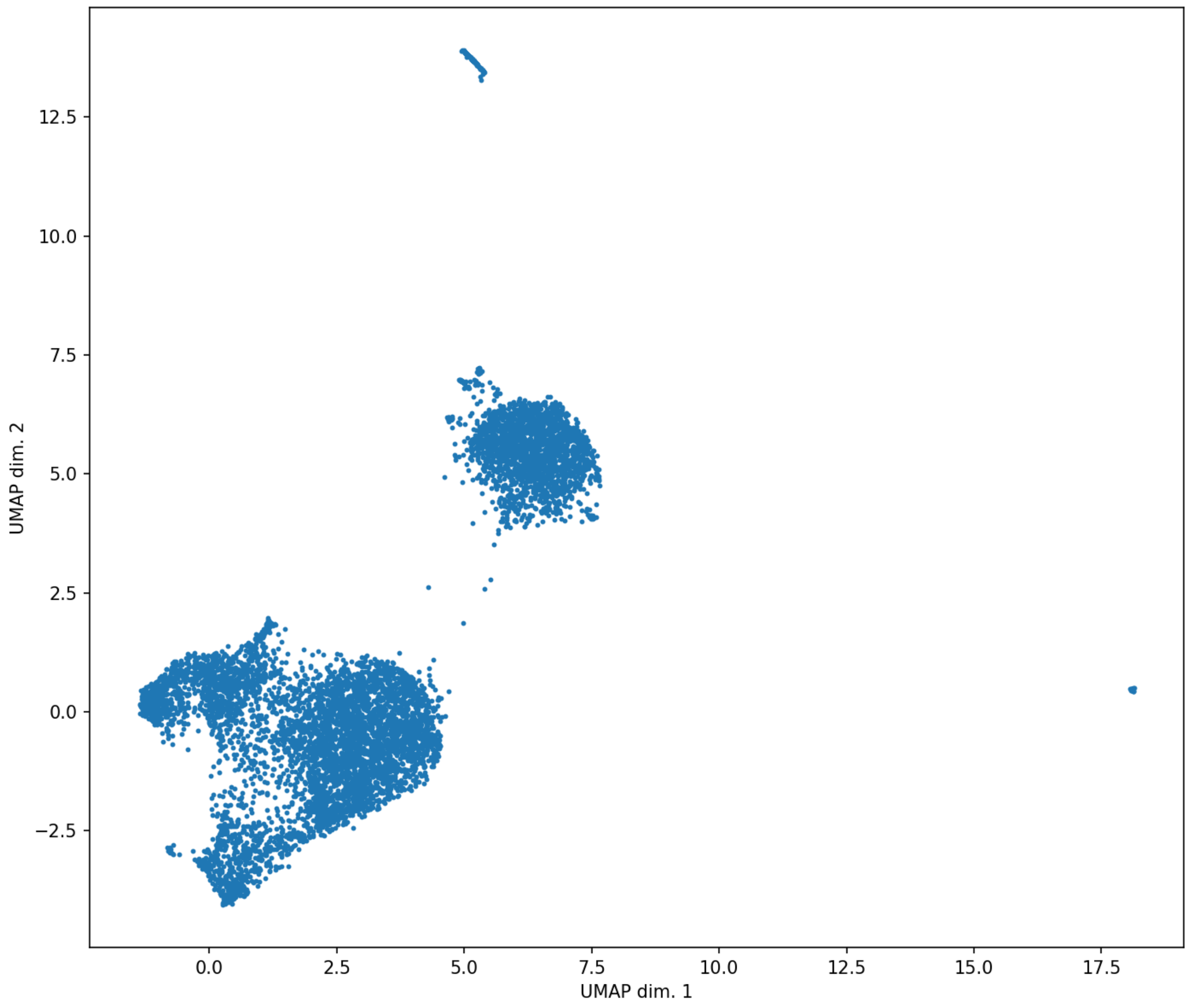

UMAP plot: each dot represents a cell

ScanpyClustering 1.0.0

Random seed: **13513**

Assay including matrix and geneIds: **Log transformed assay** (from step 5: Log transformation 1.0.0)

Cell coordinates for visualization: **PC1 vs. PC2** (from step 6: Principal Component Analysis 1.0.0)

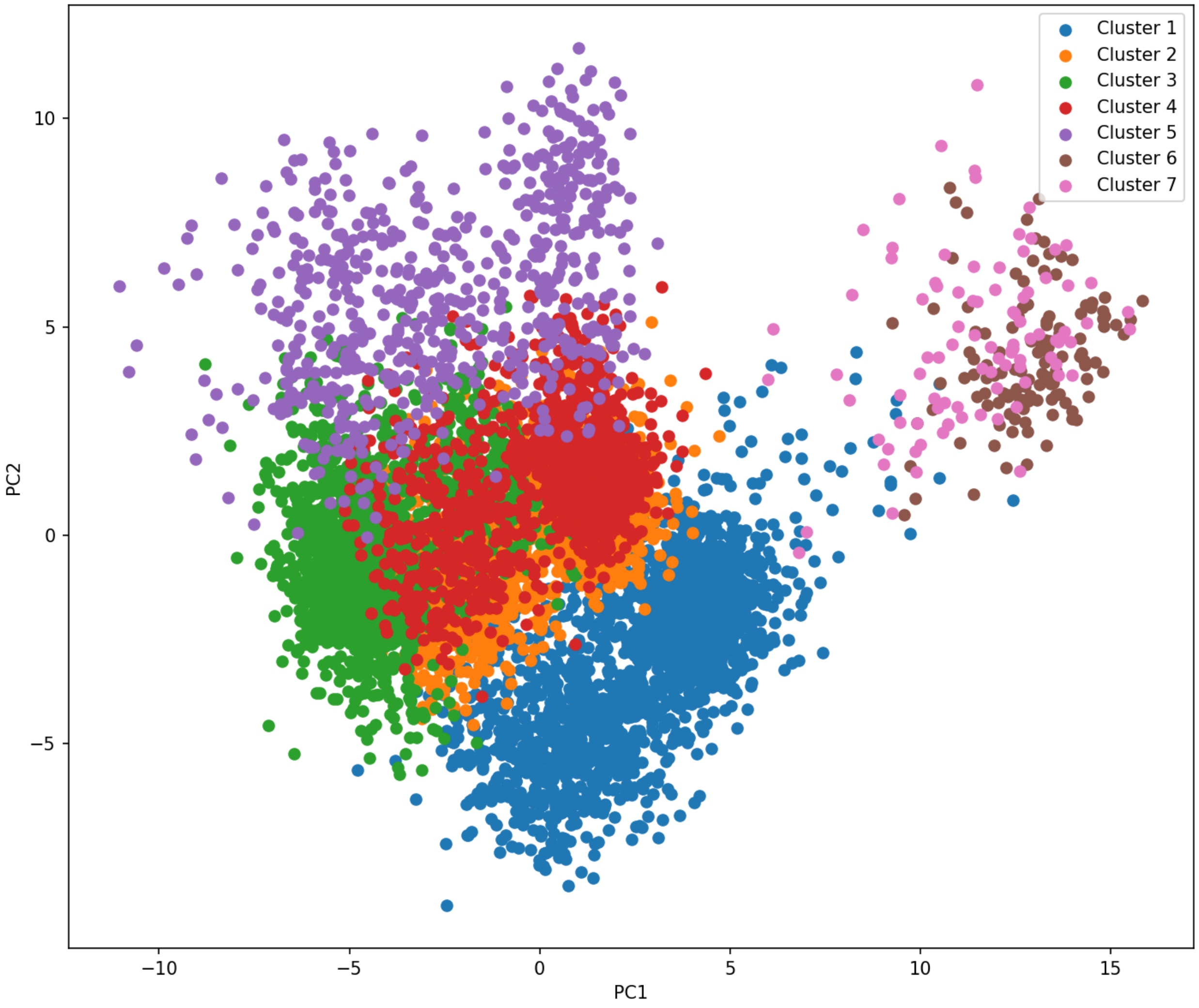

Scatter-plot using imported cell coordinates. Each dot represents a cell. The colors indicate the indentified cell clusters.

ScanpyClustering 1.0.0

Random seed: **13513**

Assay including matrix and geneIds: **Log transformed assay** (from step 5: Log transformation 1.0.0)

Cell coordinates for visualization: **t-SNE coordinates** (from step 7: t-Distributed Stochastic Neighbor Embedding 1.0.0)

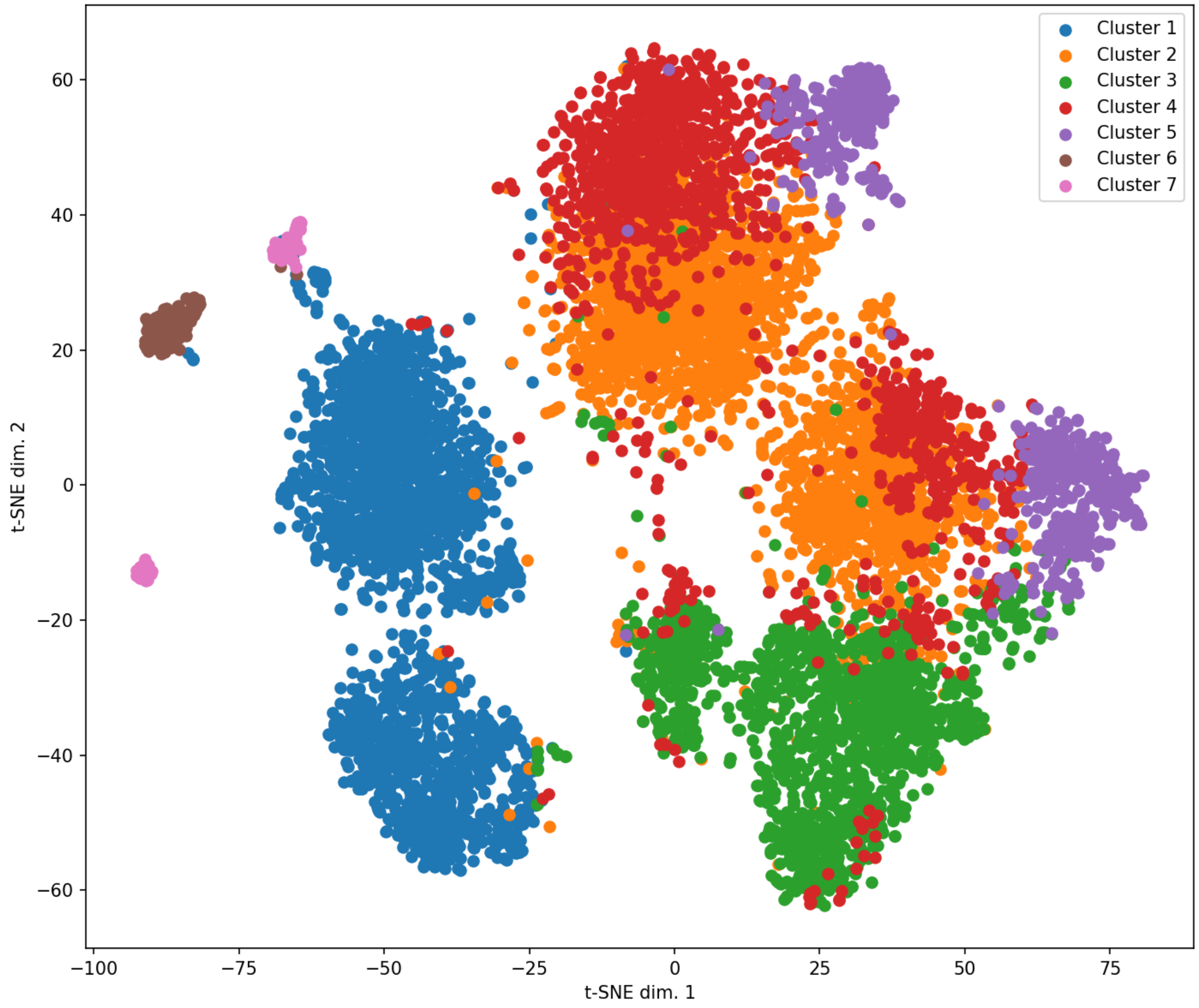

Scatter-plot using imported cell coordinates. Each dot represents a cell. The colors indicate the indentified cell clusters.

ScanpyClustering 1.0.0

Random seed: **13513**

Cell coordinates for visualization: **UMAP coordinates** (from step 8: UMAP 1.0.0)

Assay including matrix and geneIds: **Log transformed assay** (from step 5: Log transformation 1.0.0)

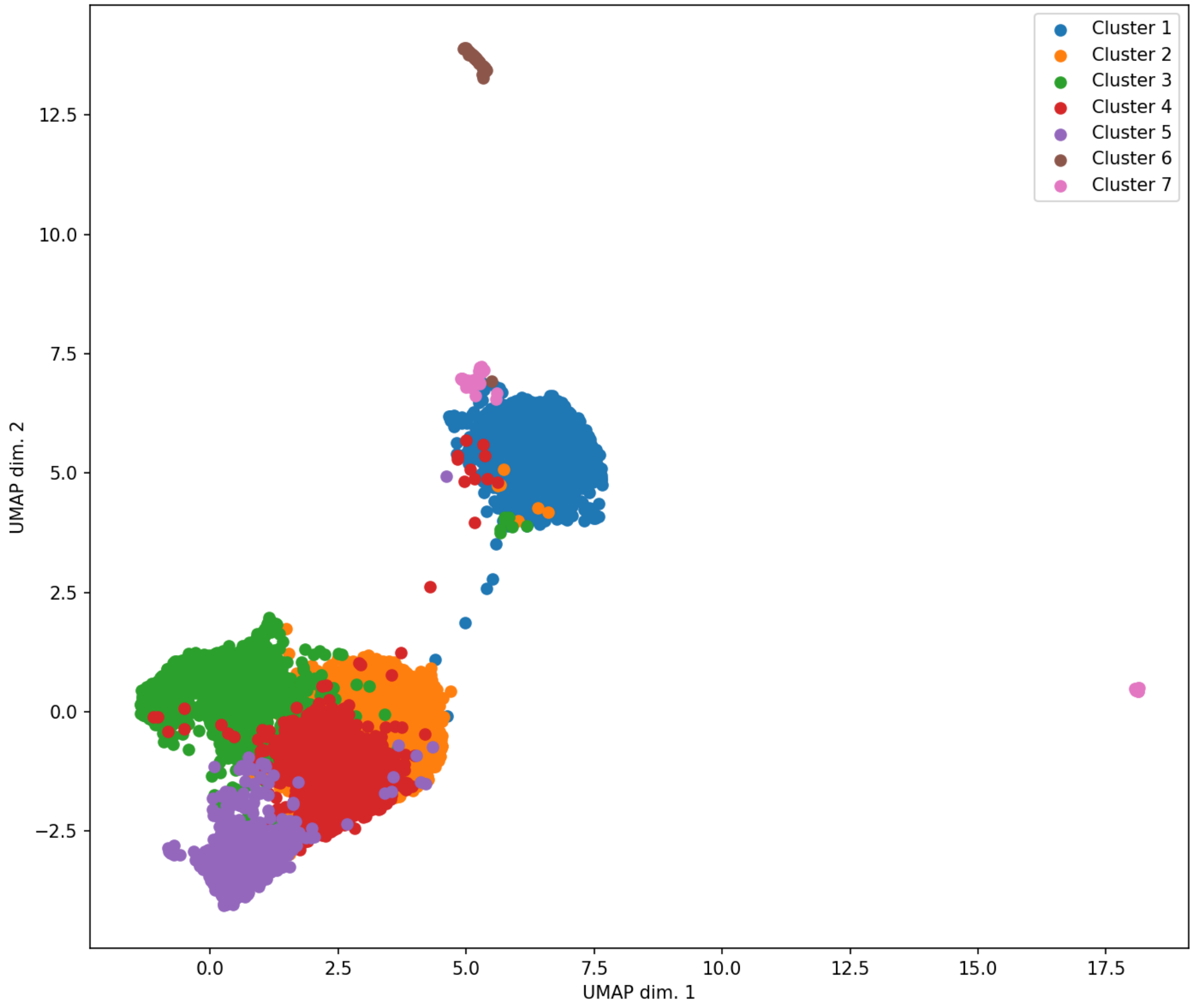

Scatter-plot using imported cell coordinates. Each dot represents a cell. The colors indicate the indentified cell clusters.

Marker Genes Identification 1.0.0

Group vector: **Cluster assignment** (from step 11: ScanpyClustering 1.0.0)

Assay including matrix and geneIds: **Log transformed assay** (from step 5: Log transformation 1.0.0)

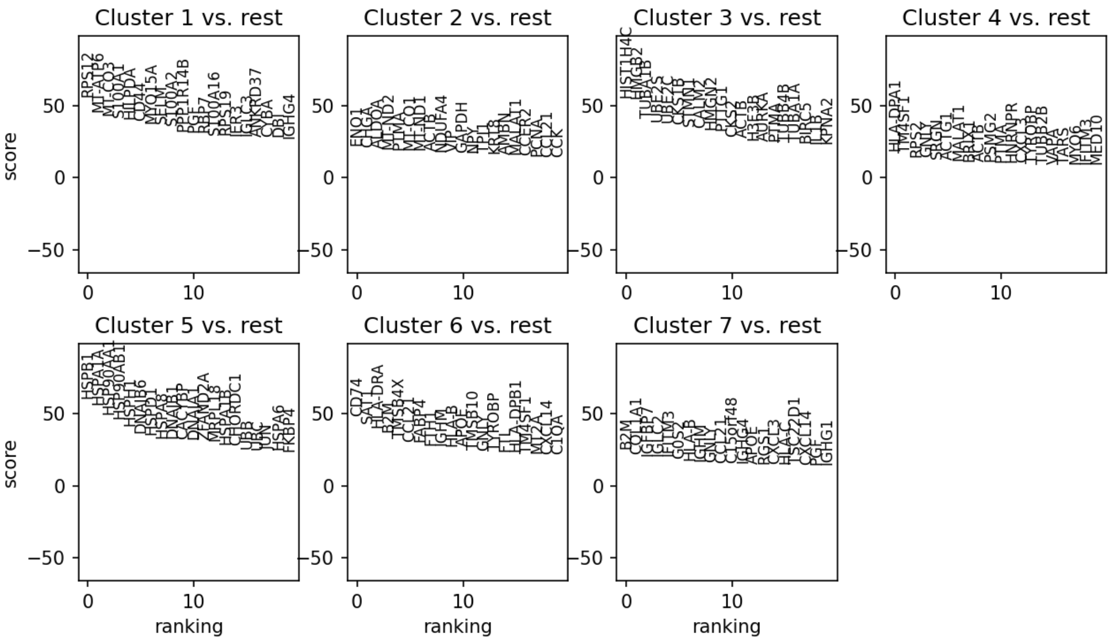

One-vs-rest marker genes

Broad GSEA 1.0.0

The species: **"human"**
The database for the enrichement analysis: **"kegg"**
Number of repeats for calculating p-values: **1000**
A list of genes with their scores (usually output from scanpy scoring for example): **Marker score (Cluster 1 vs. rest)** (from step 12: Marker Genes Identification 1.0.0)

gset_name	gset_size	nes	p_val	fdr
KEGG_ANTIGEN_PRC	30	2.4183472526	0.001	0.063
KEGG_MAPK_SIGNAI	45	2.242569416	0.005	0.105
KEGG_VIRAL_MYOCA	25	2.459714285	0.005	0.105
KEGG_PATHOGENIC_	14	2.5451885223	0.007	0.11025
KEGG_REGULATION_	25	2.3859803876	0.01	0.126
KEGG_GLYCOLYSIS_G	13	2.3783434423	0.021	0.189
KEGG_TYPE_I_DIABE	17	2.3151470657	0.021	0.189
KEGG_TIGHT_JUNCT	16	2.3476164319	0.035	0.275625
KEGG_GRAFT_VERSU	19	2.1367696417	0.047	0.2953125

Broad GSEA 1.0.0

The species: **"human"**
The database for the enrichement analysis: **"kegg"**
Number of repeats for calculating p-values: **1000**
A list of genes with their scores (usually output from scanpy scoring for example): **Marker score (Cluster 2 vs. rest)** (from step 12: Marker Genes Identification 1.0.0)

gset_name	gset_size	nes	p_val	fdr
KEGG_GLYCOLYSIS_G	13	4.2332424448	0	0
KEGG_PATHOGENIC_	14	3.6287072467	0	0
KEGG_ALZHEIMERS_	20	3.969936	0	0
KEGG_TIGHT_JUNCT	16	3.0309404996	0.004	0.063
KEGG_HUNTINGTONS	16	2.8748446055	0.009	0.1134
KEGG_REGULATION_	25	2.3360851389	0.036	0.324
KEGG_PARKINSONS_	12	2.5324904944	0.036	0.324
KEGG_UBIQUITIN_ME	12	2.3614030539	0.054	0.42525
KEGG_OOCYTE_MEIC	15	2.1520125214	0.08	0.56

Broad GSEA 1.0.0

The species: **"human"**
The database for the enrichement analysis: **"kegg"**
Number of repeats for calculating p-values: **1000**
A list of genes with their scores (usually output from scanpy scoring for example): **Marker score (Cluster 3 vs. rest)** (from step 12: Marker Genes Identification 1.0.0)

gset_name	gset_size	nes	p_val	fdr
KEGG_OOCYTE_MEIC	15	3.9142438048	0	0
KEGG_PATHOGENIC_	14	3.6276032782	0.001	0.021
KEGG_UBIQUITIN_ME	12	3.2339121482	0.001	0.021
KEGG_CELL_CYCLE	20	3.0585714514	0.004	0.063
KEGG_REGULATION_	25	2.3460916724	0.039	0.35
KEGG_ANTIGEN_PRC	30	2.2842579982	0.04	0.35
KEGG_INSULIN_SIGN	12	2.5842161078	0.05	0.35
KEGG_TIGHT_JUNCT	16	2.4147621326	0.05	0.35
KEGG_ALLOGRAFT_R	17	2.3470481376	0.06	0.35

Broad GSEA 1.0.0

The species: **"human"**
The database for the enrichement analysis: **"go"**
Number of repeats for calculating p-values: **1000**
A list of genes with their scores (usually output from scanpy scoring for example): **Marker score (Cluster 1 vs. rest)** (from step 12: Marker Genes Identification 1.0.0)

gset_name	gset_size	nes	p_val	fdr
GO_CONDENSED_CH	17	2.7298822377	0	0
GO_REGULATION_OF	139	1.9514943306	0	0
GO_RNA_BINDING	138	2.0809844395	0	0
GO_CONDENSED_CH	24	2.8174020056	0	0
GO_ESTABLISHMENT	152	1.8743619136	0	0
GO_REGULATION_OF	23	2.7517004946	0	0
GO_MITOTIC_SISTER	16	2.6126316537	0	0
GO_MICROTUBULE_C	43	2.3322495613	0	0
GO_REGULATION_OF	87	2.3645791588	0	0

Broad GSEA 1.0.0

The species: **"human"**
The database for the enrichement analysis: **"go"**
Number of repeats for calculating p-values: **1000**
A list of genes with their scores (usually output from scanpy scoring for example): **Marker score (Cluster 2 vs. rest)** (from step 12: Marker Genes Identification 1.0.0)

gset_name	gset_size	nes	p_val	fdr
GO_CALCIUM_DEPEN	12	3.4634105437	0	0
GO_CELL_PROJECTI(	139	2.3612219797	0	0
GO_GLUCOSE_METAI	21	3.4714676181	0	0
GO_OXIDATION_REDU	78	2.8874490122	0	0
GO_NEGATIVE_REGU	14	3.5490548993	0	0
GO_ACTIN_FILAMENT	50	2.7609463215	0	0
GO_PURINE_CONTAII	35	3.6790812319	0	0
GO_STRUCTURAL_C(	18	3.8285672551	0	0
GO_CARBOHYDRATE_	54	2.6840172988	0	0

Broad GSEA 1.0.0

The species: **"human"**
The database for the enrichement analysis: **"go"**
Number of repeats for calculating p-values: **1000**
A list of genes with their scores (usually output from scanpy scoring for example): **Marker score (Cluster 3 vs. rest)** (from step 12: Marker Genes Identification 1.0.0)

gset_name	gset_size	nes	p_val	fdr
GO_NEGATIVE_REGU	88	2.5290871941	0	0
GO_ADENYL_NUCLE(	110	2.4677889845	0	0
GO_POSITIVE_REGUL	17	4.0281776068	0	0
GO_ORGANELLE_FIS!	46	4.3648616611	0	0
GO_CHROMATIN_OR(	34	3.3637933508	0	0
GO_DNA_PACKAGIN(	18	3.6002319314	0	0
GO_SPINDLE_POLE	16	4.015371666	0	0
GO_CHROMATIN	38	3.2656739887	0	0
GO_POSITIVE_REGUL	83	2.5615805609	0	0

Pseudotime construction 1.0.0

Number of neighbors to calculate: **20**
Method for computing connectivities: **"gauss"**
The input assay to use: **Log transformed assay** (from step 5: Log transformation 1.0.0)

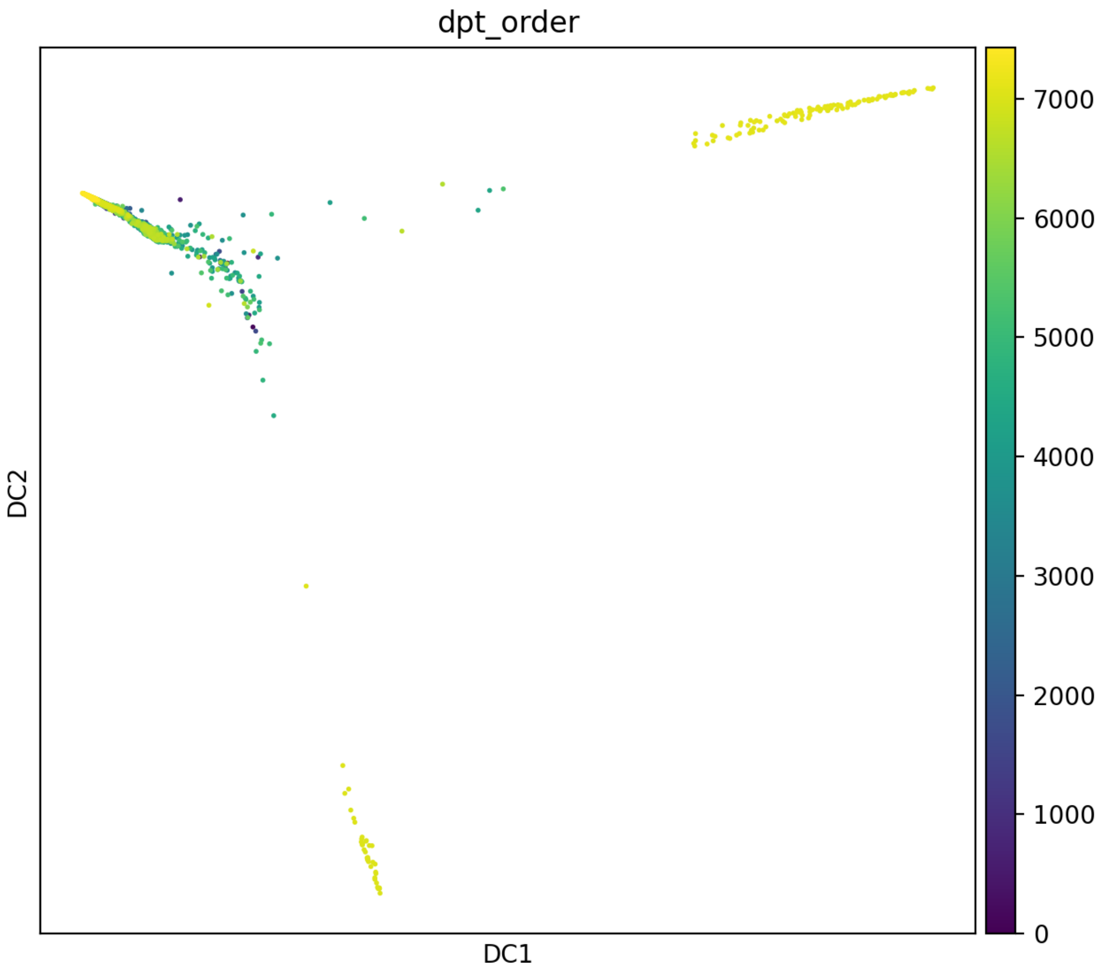

Cell order

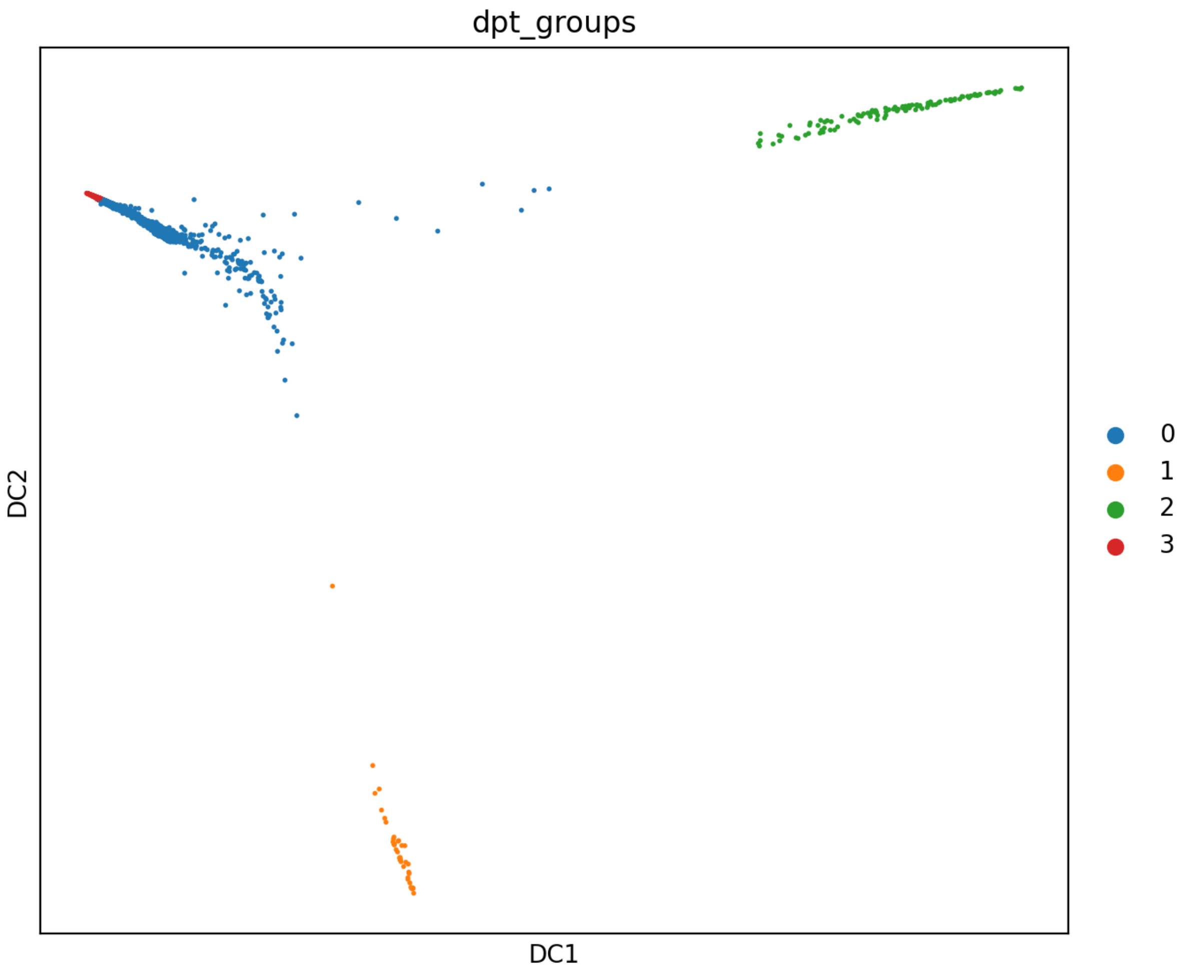

Cell groups

Use the browser back button to return to the project steps.
